# Whole Body Vibration at Different Exposure Frequencies: Infrared Thermography and Physiological Effects

**DOI:** 10.1155/2015/452657

**Published:** 2015-01-14

**Authors:** Anelise Sonza, Caroline C. Robinson, Matilde Achaval, Milton A. Zaro

**Affiliations:** ^1^Programa de Pós-Graduação em Neurociências, Instituto de Ciências Básicas da Saúde, Universidade Federal do Rio Grande do Sul, Sarmento Leite, 500, 90050-170 Porto Alegre, RS, Brazil; ^2^Programa de Pós-Graduação em Ciências da Saúde, Universidade Federal de Ciências da Saúde de Porto Alegre, Sarmento Leite, 245, 90050-170 Porto Alegre, RS, Brazil; ^3^Departamento de Ciências Morfológicas, Instituto de Ciências Básicas da Saúde, Universidade Federal do Rio Grande do Sul, Sarmento Leite, 500, 90050-170 Porto Alegre, RS, Brazil

## Abstract

The aim of this study was to investigate the effects of whole body vibration (WBV) on physiological parameters, cutaneous temperature, tactile sensitivity, and balance. Twenty-four healthy adults (25.3 ± 2.6 years) participated in four WBV sessions. They spent 15 minutes on a vibration platform in the vertical mode at four different frequencies (31, 35, 40, and 44 Hz) with 1 mm of amplitude. All variables were measured before and after WBV exposure. Pressure sensation in five anatomical regions and both feet was determined using Von Frey monofilaments. Postural sway was measured using a force plate. Cutaneous temperature was obtained with an infrared camera. WBV influences the discharge of the skin touch-pressure receptors, decreasing sensitivity at all measured frequencies and foot regions (*P* ≤ 0.05). Regarding balance, no differences were found after 20 minutes of WBV at frequencies of 31 and 35 Hz. At 40 and 44 Hz, participants showed higher anterior-posterior center of pressure (COP) velocity and length. The cutaneous temperature of the lower limbs decreased during and 10 minutes after WBV. WBV decreases touch-pressure sensitivity at all measured frequencies 10 min after exposure. This may be related to the impaired balance at higher frequencies since these variables have a role in maintaining postural stability. Vasoconstriction might explain the decreased lower limb temperature.

## 1. Introduction

Vibration induces physiological effects that are strongly influenced by parameters such as vibration frequency, amplitude, duration, and direction of exposure. Vibratory stimuli interact in a complex manner in the human body and cause physiological effects changing balance [1–3], cutaneous sensitivity [4], muscle activation [5], and blood flow [6], among others. In the ergonomic field, vibration is widely reported to produce motor disorders [7], loss of sensitivity, decreased blood flow, and pathologies such as vibration white finger or carpal tunnel syndrome [8].

Whole body vibration (WBV) training platforms have been considered an exercise modality and the reduced effort required with their use might explain their popularity [9, 10]. The variety of WBV platform settings (vibration mode, frequency, amplitude, and exposure duration) that have been used in the available studies and the controversial findings may, in part, explain the inexistence of training standards [10–13]. An understanding of its acute effects and studies with a wider range of platform settings might help elucidate this question. A better understanding of the parameter settings might also help health area professionals to choose the most suitable settings for specific rehabilitation conditions, sports training, and normal subjects.

The skin is the body's largest organ. The dermis comprises a dense network of mechanoreceptors, in addition to nerve endings that penetrate the epidermis and together provide the senses of touch, pressure, vibration, pain, and heat. It works as the body's thermoregulator, controlling blood flow within a few millimeters of the body surface and aids the sense of balance to modulate posture and gait.

The influence of WBV on large diameter fibers has been shown directly after WBV for touch-pressure sensitivity [4, 14] and influences the discharge of the skin's fast adapting receptors at 30 Hz [4] (Meissner's corpuscles) and 200 Hz [3, 4] (Pacinian corpuscles) causing reduced sensitivity. The presence of postvibratory disturbances affecting skin mechanoreceptive afferent units seems to be a unanimity among studies using different WBV settings. It is well reported that reduced sensitivity in the mechanoreceptors might increase the risk of falls [15, 16] because they are part of the sensory feedback system that provides balance [16, 17]. However, previous studies investigating the acute effects of WBV on balance have provided controversial results with the use of different platform settings. Studies have shown balance enhancement [3], nonsignificant results [1, 14], and a reduction in overall postural stability [2]. Hence, it is important to confirm the association between balance control and the reduction in sensitivity after exposure to different frequencies of WBV.

Additionally, thermal measurements provide information related to total blood flow function and it is interesting to understand the effects of WBV on skin temperature. A study with thermography showed an increase in temperature of the medial gastrocnemius with short intercalary exposures [18]. However, to the best of our knowledge, no previous study has demonstrated the periphery thermal effects during the whole WBV session and in the subsequent minutes. It is interesting to verify the temperature of the lower limbs after the end of WBV to see whether the peripheral thermal pattern persists during the preselected time period (10 min).

Another important aspect to consider is the fact that no study has investigated the combination between several physiological parameters at different frequencies of exposure. Therefore, the purpose of this study was to investigate the effects of WBV on different physiological variables and at different WBV settings. The specific hypotheses were (A) cutaneous sensitivity to touch pressure at different frequencies of exposure decreases after WBV; (B) lower limb temperature might increase after WBV; (C) balance might decrease after WBV.

## 2. Methods

This was an experimental before-and-after study investigating the acute effects of 15 minutes of WBV at four different vibration frequencies on healthy young individuals, each individual being their own comparator. The study took place at the Biomechanics Laboratory of the Brazilian Institute of Leather, Shoe and Artefacts Technology (*Instituto Brasileiro de Tecnologia do Couro, Calçados e Artefatos*, IBTeC), Novo Hamburgo, Rio Grande do Sul, Brazil, from May 2012 to May 2013.

### 2.1. Participants

Participants were invited from the community. Those eligible were healthy young adults of both genders, aged from 18 to 35 years of age. Exclusion criteria were muscle-skeletal disorders, cognitive or physical dysfunction and the contra-indications for WBV previously reported by Bautmans et al. (2005) [19]. All measurements were approved by the Ethical Committee of the Federal University of Rio Grande do Sul, Brazil (protocol number 22626) and were in accordance with the Declaration of Helsinki.

#### 2.1.1. Sample Size Definition

As this study was designed considering the particularities of using thermography to detect a before-and-after difference in lower limb temperature of 1.5 (0.9)°C [20], with a two-sided 5% significance level and a power of 90%, a minimum sample size of 20 participants was necessary, given an anticipated dropout rate of 10%. As sample determination did not consider the other measured outcomes, a statistical power was calculated and presented for each analysis.

### 2.2. Procedures

**Figure 1:**
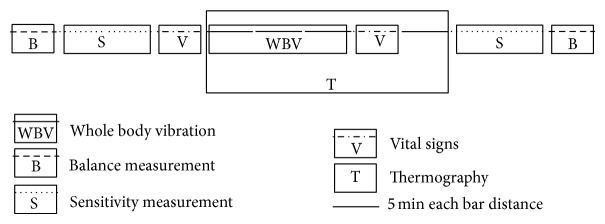
Protocol timeline for one single session. Each bar distance corresponds to 5 minutes in the protocol.

All the procedures were conducted in the same room with controlled air humidity (50 ± 5%) and temperature (21 ± 2°C). After assessing the participants' characteristics, data collection included the assessment of the foot sensitivity for touch-pressure sensitivity, balance, and physiologic and thermographic measurements, before and after exposure to WBV for 15 minutes at each of the four different vibration frequencies. Each session of exposure to a vibration frequency and the respective before-and-after assessments lasted approximately one hour. They were performed at intervals of 48 hours to avoid the cumulative effects of vibration. For each subject the protocol extended for 10 days. To be included in the analysis, each participant must have attended four visits to the Biomechanics Laboratory. During each session, the measurements were taken in the following sequence: (a) cutaneous sensitivity (10 min); (b) balance (5 min); (c) vital signs (5 min); (d) IR thermography during 15 minutes of WBV exposure and during the 10 minutes immediately following WBV; (e) vital signs, (f) cutaneous sensitivity; (e) balance. The sequence of measurements taken in a single session is presented in the protocol timeline (Figure 1).

WBV exposure was performed on a vibration platform (Pro-Form Bio-Vibe, Icon Fitness, Colorado, USA) with allowing only four frequency settings (named *f*_1_, *f*_2_, *f*_3_, and *f*_4_, resp.) at a fixed vibration displacement in a vertical synchronous vibration. Thus, the choice of these vibration parameters was determined due to the limitations of the equipment. The accuracy of the frequencies was measured using a uniaxial piezoelectric accelerometer, model DeltaTron 4507 B 006A (Brüel & Kjær, Denmark), and dedicated software and values of 31 (*f*_1_), 35 (*f*_2_), 40 (*f*_3_), and 44 (*f*_4_) Hz were found. To measure the amplitude of the WBV machine, a 50 kg weight with a reflexive marker attached was placed on the platform while the measurements were taken. The Spica Tek motion analysis system (Spica Technologies, New Hampshire, USA) with a video camera, model IPX VGA 210-L (Imperx, Florida, USA) at a sample frequency of 200 Hz was used to register the displacement of the marker. A peak-to-peak amplitude of approximately 1 mm was found at all the tested frequencies. As the participants performed only one of the four vibration frequencies at each session, the frequency order was randomly selected. During WBV exposure, participants were static, standing barefoot on a 3 mm ethylene vinyl acetate (EVA) foam, fixed to the platform base to avoid foot slip, with legs slightly bent (knee flexed approximately 20°, considering a full knee extension of 0°) with feet shoulder-width apart, without upper-limb support. The participants maintained this position for 15 minutes during each WBV exposure. During the 10 minutes following WBV exposure, the participant maintained the same position on the platform but was allowed to extend the knees to avoid fatigue.

#### 2.2.1. Vital Signs

Physiological measurements such as blood pressure, axillary temperature, and respiratory and heart rates were taken before and immediately after 15 minutes of WBV exposure. For these measurements, the subjects stood barefoot on the vibration platform, without vibration, with both feet flat on the platform in a quiet environment. The subject was expected to be comfortable, relaxed, and with a recently emptied bladder. To measure blood pressure, a manually calibrated sphygmomanometer (Premium NML-105, Inmetro, Brazil) and a stethoscope (3M Littmann Classic II S.E. Stethoscope, USA) were used. The left arm was extended and the forearm was supported by the assessor at the level of the subject's heart with the palm of the hand facing up. A digital thermometer OMRON (MC-245, Japan) was used to take the axillary temperature. To measure the heart rate, the pulse was located by lightly pressing the index and middle fingers slightly to the radial side of the subject's wrist (where the radial artery is located). The heartbeats were counted in one minute (bpm). One respiratory cycle was considered to include one complete rise and fall of the chest or the inhalation and exhalation of air. To obtain a more reliable respiratory rate count, the subjects were induced to believe the count was initiated before the valid count so that they were unaware their breathing was being monitored at the time of the count. The respiratory rate was registered in one minute of breathing (rpm). All the vital signs were performed by the same assessor.

#### 2.2.2. Touch-Pressure Sensitivity Assessment

To obtain the touch-pressure sensitivity, Semmes-Weinstein monofilaments (Touch-Test 20 Piece Kit, Stoelting Co, Wood Dale, USA) were used. Before and 10 minutes after WBV exposure, touch-pressure sensitivity was measured in five anatomical regions of the right and left foot (heel, middle foot, 1st and 5th metatarsal head and hallux), following the same procedures presented by Sonza et al. (2013) [4]. The sequence in which the feet and the respective regions were measured was randomized to avoid any time lag effect on the feet evaluation. A modified 4, 2, 1 stepping algorithm proposed by Dick et al. (1993) [21], was used for the protocol and the same assessor performed these measurements before and after each WBV exposure. Because temperature influences sensitivity, foot temperature was monitored using a digital thermometer probe HT 208 (Icel, Manaus, Brazil) and maintained between 25°C and 32°C. The temperature of each foot was taken prior to measuring touch-pressure sensitivity. Those participants in whom the temperature of the foot had changed more than 2°C from baseline would be excluded from the analysis. In this study, only one subject was excluded.

#### 2.2.3. Balance Assessment

An OR6-5 force plate (AMTI, Massachusetts, USA) was used to assess balance by center of pressure (COP) sway (displacement, velocity, and area). The force platform was embedded in the floor. The balance was measured before WBV exposure and 20 minutes after the end of the 15 minutes of WBV exposure, immediately following the touch-pressure sensitivity assessment. In each trial, the subject stood barefoot, in a single leg stance, with eyes open for one minute. Participants were verbally instructed to look straight ahead with their body erect and hands on the waist and to keep balance. Two trials were performed on alternate legs, with a one-minute interval between trials to avoid fatigue. The order of the first foot was sorted at random. Balance assessments were conducted by the same assessor.

A time domain analysis was performed to obtain summary measures of the COP values in both the anterior-posterior (A-P) and medial-lateral (M-L) directions: (a) resultant mean COP velocity; (b) COP length; (c) COP ellipse area. The AP and ML components of COP velocity were calculated by the root mean square of the vector of the AP and ML components of the instantaneous velocity of the COP. The COP length was considered the total displacement of the body's COP during data collection. Although the mean COP velocity and length are mathematically equivalent [22], we decided to keep the values of both measurements in order to allow further comparisons. The 95% confidence ellipse area was calculated using the principal component analysis [23]. COP analysis algorithms [24] were implemented in MATLAB 7.9 (Mathworks Inc., Natick, USA). The COP data were low-pass filtered at 5 Hz with a fourth-order and zero-lag Butterworth filter [22], since most of the power of the signal was below 2 Hz [25]. The first 10 seconds of data at the beginning of each trial were discarded and hence not considered in determining the sway measures. Thus, the maximum duration of sway was 50 seconds.

#### 2.2.4. Lower Limb Temperature Assessment

The temperature of the lower limbs was assessed using infrared thermography. A PV-320T (Electrophysics, New Jersey, USA) thermal imaging camera (resolution of 320 × 240 pixels) was used to obtain thermal imagery in the 3 to 14 *μ*m spectral range, sensitivity of 0.08°C with a maximum error of 2%. Images were captured and processed using Electrophysics Velocity 2.4 (Electrophysics, New Jersey, USA) image analysis software with automatic calibration and emissivity of 0.971. The camera was placed at an angle of approximately 70° to the surface, 0.7 m above the floor and 2.5 m from the vibration platform to provide a full view of the lower limbs. The infrared imaging protocol followed the recommendations of the American Academy of Thermology [26]. A sequence of images of the dorsal part of the lower limbs was automatically recorded for 25 minutes, 1 frame per second (FPS). The vibration platform was turned on for the first 15 minutes and turned off for the last 10 minutes.

The mean data points within a square drawn in the central area of the thigh, knee, lower leg, and foot were analyzed as regions of interest [27] set by the same assessor. Before the thermographic assessment, the participants remained with the lower limbs bared for 20 minutes in order to equilibrate their body-surface temperature to the room temperature. The lower limbs made no contact with any surface during this period or during the 25 minutes the subject spent on the platform. The same clothes were used in the four WBV exposures.

### 2.3. Statistical Analysis

The mean values and the standard error (±SE) were calculated for all the participants and all analyzed outcome measurements. The normality of the data distribution was assessed using the Shapiro Wilks test. Despite the nonparametrical nature of the data, a parametric test was chosen because a nonparametric test for many factors and analysis increases the chances of a type I error [28, 29]. Moreover, with small sample sizes, nonparametric analyses are less likely to detect effects, or the power is reduced, or the confidence intervals are wider. Differences between the measurements obtained before and after WBV exposure were analyzed using the MANOVA multivariate analysis of variance test for multiple dependent variables (frequency, time before versus after, right and left sides, different regions of the body for the sensitivity and thermographic data, and balance variables). To reduce the effects of nonuniformity in the data distribution, all values were transformed using a logarithmic (base 10) transformation. COP data for only 17 of the original 24 participants were analyzed because some data were missing from the digital recording. For the thermographic frames sequence, the mean temperature in each frame was used to obtain 25 data points, representing every minute from the sequence. A paired *t*-test was applied to compare the sequences minute by minute. The adopted level of significance was *α* = 0.05 and the type I error was controlled by the observed power (1-beta > 0.8).

## 3. Results

A total of 24 (26.4 ± 4.1 years; 11 male, 13 female) participants, with mean height 170.5 (±8.6) cm and weight of 66 (±12.6) kg, participated in the study. None of the participants had trained on vibration platforms prior to the experimental interventions.

### 3.1. Vital Signs

The MANOVA analysis for all subjects, for the physiological measurements (blood pressure, axillary temperature, respiratory, and cardiac rates), including frequency and time (before versus after), showed effects of time (*P* < 0.01; power = 0.87) on heart rate.  Table 1 shows the results for all 24 subjects comparing the conditions “before and after” WBV.

No effects were found between the measured frequencies. Heart rate was higher at 31 Hz, comparing before and after WBV (*P* < 0.01).

### 3.2. Touch-Pressure Sensitivity

Baseline plantar sensitivity presented levels consistent with normality, since, under the normal conditions, the values for pressure sensation were between 3.08 and 3.59 according to the Semmes-Weinstein monofilament (Table 2).

**Figure 2:**
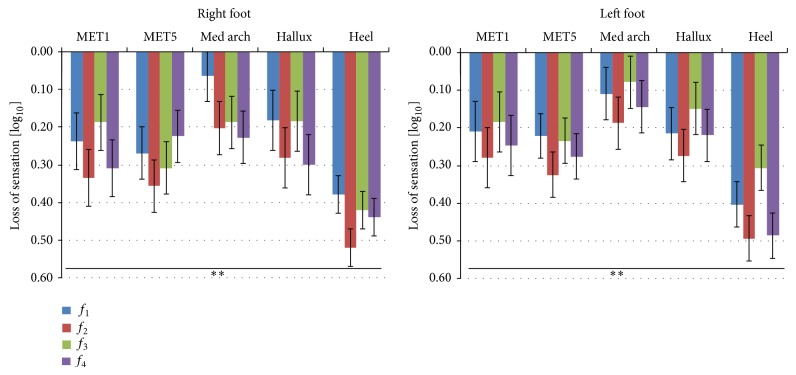
Loss of sensation (LOS) and standard error (±SE) in touch-pressure sensitivity with respect to the control condition, for both feet, five regions (heel, medial arch, 5th metatarsal head, 1st metatarsal head, and hallux). All 24 subjects were measured 10 minutes after WBV exposure, at 4 different frequencies of WBV (*f*_1_ = 31, *f*_2_ = 35, *f*_3_ = 40, and *f*_4_ = 44 Hz) (^**^ *P* < 0.01).

A significant loss of sensation intraregions was found after 15 minutes of WBV exposure compared to baseline (time effect; *P* < 0.01; power = 1.0) for touch-pressure sensitivity in all five tested regions of the right and left foot (Figure 2).

There were no differences comparing interregions between the right and left foot regarding time or frequency effects.

### 3.3. Balance

**Figure 3:**
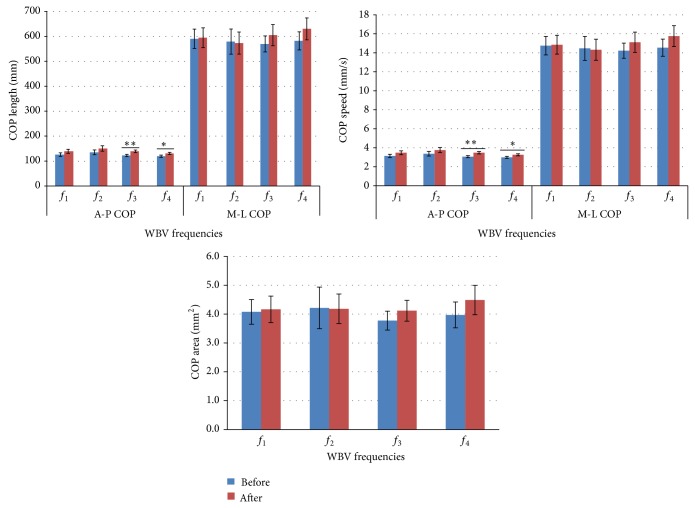
Mean and standard error values of the COP summary measures during the 60-second single leg stance trials for the different frequency WBV groups, before and 20 minutes after WBV intervention. *f*_1_ = 31 Hz; *f*_2_ = 35 Hz; *f*_3_ = 40 Hz; *f*_4_ = 44 Hz (*n* = 17) (^*^ *P* < 0.05; ^**^ *P* < 0.01).

Considering the postural stability data, the means and standard errors of COP values for each WBV frequency are presented in Figure 3. The measurements were taken 20 minutes after WBV exposure. In the A-P direction, regarding COP velocity and COP length, there was a significant change in the comparison between before and after WBV for the higher frequencies. For *f*_3_ and *f*_4_, the participants showed a higher A-P COP velocity (*f*_3_ before versus after: *F* = 11.67, *P* < 0.01; *f*_4_ before versus after: *F* = 5.42, *P* = 0.023) and for A-P COP length as well (*f*_3_ before versus after: *F* = 11.9, *P* < 0.01; *f*_4_ before versus after: *F* = 6.5, *P* = 0.013). No differences in balance were found within the *f*_1_ and *f*_2_ frequencies for any of the analyzed COP variables. Regarding the COP area and the M-L COP velocity and length, no differences were found when comparing the conditions before versus after WBV exposure.

The analysis including frequencies, lower limbs (right versus left), and time (before versus after) showed effects for frequency (*P* < 0.01; power = 0.99) and time (*P* < 0.01; power = 0.95). No effect was found between the right and left lower limbs (*P* = 0.09; power = 0.79).

### 3.4. Lower Limb Temperature

**Figure 4:**
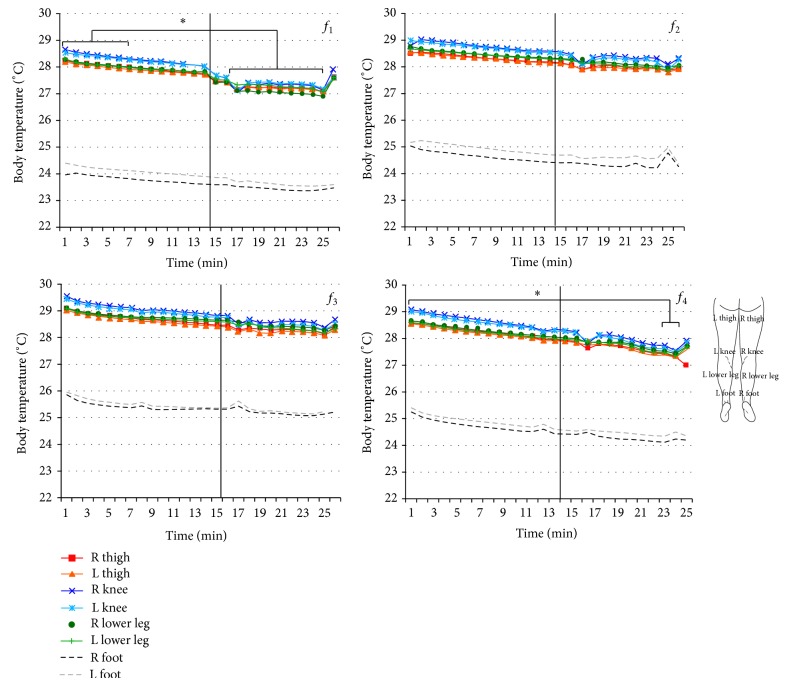
Thermograph curve from the posterior of the lower limbs obtained during 15 minutes of exposure and 10 minutes after exposure to four different WBV frequencies, in four regions of the right and left limbs (thigh, knee, lower leg, and foot). *f*_1_ = 31 Hz; *f*_2_ = 35 Hz; *f*_3_ = 40 Hz; *f*_4_ = 44 Hz (*n* = 24) (^*^ *P* < 0.05).

Infrared thermography was used to determine the temperature response to WBV in the lower limbs. The temperature values obtained during the 15 minutes of and 10 minutes following WBV exposure for each frequency vibration are plotted in Figure 4. In general, a decrease was observed in lower limb temperature during the 15 minutes of and 10 minutes following WBV exposure. A slight increase in the temperature was observed at the end of the recovery time, which is an indication that the temperature had started to return to the baseline condition.

Comparing lower limb temperature in the 1st minute and at the 25th minute, the MANOVA analysis showed effects for frequency (*P* < 0.01; power = 1.0) and time (*P* < 0.01; power = 1.0). The pairwise comparison showed a significant difference (*P* < 0.01) for the regions of the thigh, knee, and lower leg between time and frequencies. For the foot region, there was no difference between the 1st and 25th minute (*P* = 0.87). However, there were differences between the frequencies.

The comparison between *f*_1_ and *f*_3_ showed differences in skin temperature between all the regions of the lower limbs (*P* < 0.01). Comparing *f*_3_ versus *f*_4_ there were differences in the regions of the thigh (*P* = 0.001) and lower leg (*P* = 0.013). For the regions of the foot, *f*_1_ presented differences between the 1st and 25th minute in comparison to the other three analyzed frequencies. Comparing *f*_2_ versus *f*_3_, there were also differences in the same region (foot) (*P* = 0.005).

A *t*-test was applied to compare the minute-by-minute mean temperatures in the posterior region of the lower limbs (Figure 4). A significant decrease in temperature was found between the first 7 minutes in comparison to the last minutes (from 16 to 24) for the regions of the thigh, knee, and lower limbs at *f*_1_. At *f*_4_, a significant decrease in lower limb temperature was found between the 1st minute and minutes 22 to 24 for the regions of the thigh and knee and compared to the last minute for the lower leg. In the region of the foot in general, at frequencies of 35 and 40 Hz, there was no significant decrease in the temperature (*P* < 0.05).

## 4. Discussion

The results of this study support the understanding that, for young healthy people, in general, a single 15-minute session of WBV does not affect physiological variables such as blood pressure, axillary temperature, or the respiratory and heart rates. These findings are in agreement with a study [30] that showed that the cardiovascular effects of WBV performed to exhaustion are mild compared to those of bicycle ergometry. Although WBV exercise has been considered an exercise modality, when compared with traditional aerobic exercises such as cycling, walking, and dancing, its characteristics are different in relation to responses to the same variables.

A reduction in the touch-pressure sensitivity of the feet at different frequencies of the vibration platform was found 10 minutes after the use of the device. Using different WBV settings from those used in the present study, the authors [3, 4, 14] reported the same results for cutaneous sensitivity immediately after WBV. Sonza et al. [4] found that the recovery time for the right foot and the same foot locations for touch-pressure sensitivity was less than 1 hour. For vibration sensitivity, the recovery time was about 2.5 hours after a single WBV session. Considering studies with different frequencies, vibration modes, and amplitudes, cutaneous sensitivity always seems to decrease immediately after WBV.

The central nervous system uses plantar cutaneous-muscular and ankle spindle afferent inputs that influence balance control during quiet and perturbed stance [31]. Balance is required for safe functional mobility, and any reduction in sensitivity might increase the likelihood of falling [4]. However, interestingly, some studies involving young healthy subjects showed acute enhancement in neuromuscular performance such as balance control [3] and muscle strength [32] or nonsignificant results [1, 14] after WBV. A study modulating lower limb somatosensory information by tendon and plantar cutaneous-muscular vibrations showed altered postural responses that increased COP displacement [31]. Regarding the balance control variables, A-P COP velocity and length, when measured 20 minutes after WBV, this study shows there is a significant decrease in balance control at the highest analyzed frequencies (40 and 44 Hz). Impaired balance may be due to decreased sensitivity resulting from disturbances in the discharge of the skin mechanoreceptors (sensory feedback system). No significant results were found either for the COP ellipse area or at the lowest frequencies. Dickin et al. [2] measured balance on a stable support surface and with opened eyes after 4 minutes of WBV during separate trials at frequencies of 10, 30, and 50 Hz. They found an increase in COP sway immediately after WBV that returned to baseline at the 10- and 20-minute postvibration assessments. Two major differences between the current study and that of Dickin et. al. (2012) [2] are the length of exposure and the postural assessment; in the former case, unipodal balance was assessed for 15 minutes, while in the latter, bipodal balance was assessed for 4 minutes. The study involving healthy subjects conducted by Schlee et al. [3] measured balance at a frequency of 27 Hz and 2 mm horizontal amplitude after 4 minutes of WBV and found an improvement in balance control immediately after WBV. The present study, in agreement with Dickin et al. [2], found no differences in balance control with the vibration platform set at 31 Hz, measured 20 minutes after vibration.

It is well documented that thermal measurements provide information related to total blood flow function. Although an increase in lower limb temperature was expected at all the studied frequencies, a decrease in lower limb temperature was found during the 15 minutes of exposure.

A delay was observed in the recovery of lower leg temperature to the baseline condition. At all frequencies, recovery began to occur at around 10 minutes after vibration, and this is particularly clear at 31 Hz. This finding is consistent with those of previous investigations [33–35] inducing hand-arm vibration. In 12 healthy participants, Olsen (1993) [36] studied the cold-induced vasoconstrictor response in the digital arteries exposed to unilateral vibration at a frequency of 31.5 Hz. It was found that 30 minutes of exposure to such vibration caused a significant increase in cold-induced arterial responsiveness in both the vibrated and nonvibrated fingers 60 minutes after the end of exposure to vibration. Some authors [35, 37] conclude that the higher the magnitudes of vibration, the greater the vasoconstriction in both the vibrated and nonvibrated fingers during recovery, which is consistent with a greater sympathetic response.

**Figure 5:**
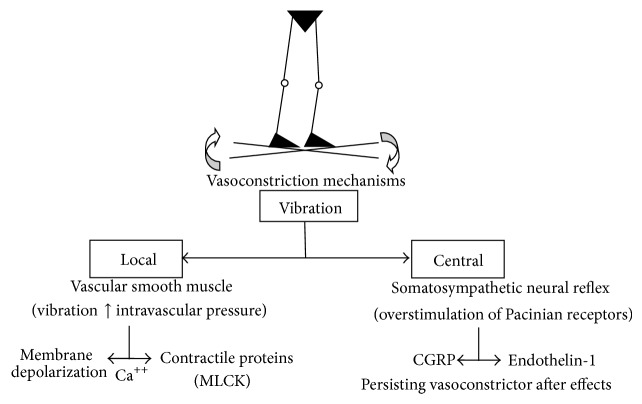
Putative local and central mechanisms involved in vascular smooth muscle vasoconstriction mediated by vibration stimulation (see text for details).

Vasoconstriction induced by vibration may be related to local and central mechanisms (Figure 5). It has been reported that threshold concentrations of the vasopeptide endothelin-1 (ET1) can sensitize the blood vessel wall to the vasoconstrictor effects of substances released by the adrenergic nerves [34]. Literature reviews implicate activation of the somatosympathetic pathway by the Pacinian vibroreceptors as the reflex mechanism producing neural activation of vasoconstriction [38, 39]. The nervous conduction from the mechanoreceptors travels along large diameter afferent *A*_*β*_ sensory fibers. The significant decrease in touch-pressure sensitivity found in the present study is evidence of somatosympathetic pathway activation through vibration. A previous study by Sonza et al. [4] found that after 10 min WBV it takes about 2.5 hours for the Pacinian vibroreceptors to recover and normalize their discharge rate. The main pathophysiological mechanism is probably an imbalance between ET1 and the calcitonin-gene-related peptide (CGRP), a powerful vasodilator present in digital cutaneous perivascular nerves [40].

Nevertheless, these thermographic results cannot exclude the possibility that local vasoconstrictor mechanisms are acting. Because the resting potential of smooth muscle is determined to a large extent by K^+^ [41], stretch-induced depolarization could be explained by activation of mechanosensitive ion channels that promote Na^+^ or Ca^+2^ influx, Cl^−^ efflux or inhibit K^+^ efflux [42]. Increasing intracellular [Ca^2+^] via IP_3_ (inositol-3-phosphate) gated channels in the sarcoplasmic reticulum sensitizes the smooth muscle apparatus to calcium, activation of myosin light chain kinase, and increases phosphorylation of the regulatory myosin light chains and thus leads to contraction [43–45]. The calcium-dependent enzyme myosin light chain kinase (MLCK) is responsible for initiating physiological contraction in smooth muscle [46] and the release of Ca^2+^ increases MLCK activation [45].

One implication of these results would suggest that the observed after effects of vibration might be interpreted as resulting from a vibration-induced vasoconstrictor response observed through the temperature decrease in the vessels of the lower legs. Vasoconstriction may have been mediated by the sympathetic nervous system and locally amplified by smooth muscle membrane depolarization and endothelium-derived contracting factors [33] in response to various chemical and physical stimuli [47].

Controversially, a study using IR thermography and different parameter settings on a vibration platform showed an increase in temperature of the medial head of the gastrocnemius [18]. In that case, as the exposure was short and intercalary, the central vasoconstriction mechanisms may not have interacted.

Considering the limits of this study, a recent paper from Alizadeh-Meghrazi et al. (2014) [48] suggests that subjects with different body mass indexes might receive different vibrations. We did not check the platform settings with all the subjects individually. The methods chosen to measure the vital signs may represent another limitation, because they may not have been sufficiently accurate to detect differences in the selected variables, in contrast to those used to measure the peripheral temperature and balance.

## 5. Conclusion

A significant loss of touch-pressure sensation was found on the sole of the right and left foot 10 minutes after the WBV session. As mechanoreceptor sensitivity is an important part of the feedback system that provides balance, the loss of balance reported here may be related to the reduced sensitivity found in this study. At the highest frequencies (40 and 44 Hz), a significant loss of balance was found for the A-P COP velocity and length variables. To avoid the risk of falls, it is important to give special attention to elderly people immediately after their use of WBV machines.

During and 10 minutes after WBV exposure, lower limb temperature decreased, probably due to vasoconstriction.

## Figures and Tables

**Table 1:** Mean and standard deviation (±SD) for all 24 subjects before and after 15 minutes of WBV exposure. The values are for physiological measurements such as blood pressure, axillary temperature, heart rate, and respiratory rate, measured at 4 different frequencies of WBV (*f*_1_ = 31, *f*_2_ = 35, *f*_3_ = 40, and *f*_4_ = 44 Hz) (^**^ *P* < 0.01).

	Systolic pressure (mmHg)		Diastolic pressure (mmHg)		Axillary temperature (°C)		Heart rate (bpm)		Respiratory rate (rmp)	
	Before	After	Before	After	Before	After	Before	After	Before	After
*f*_1_	114.3	114.2	75.7	74.9	36.1	36.1	80.3^**^	87.0^**^	15.0	16.2
SD	(±14.9)	(±16.9)	(±7.9)	(±9.2)	(±0.4)	(±0.5)	(±13.4)	(±15.3)	(±3.3)	(±3.4)
*f*_2_	113.3	112.8	75.8	75.5	36.0	34.8	80.4	85.3	15.4	16.7
SD	(±12.6)	(±14.7)	(±8.3)	(±8.4)	(±0.6)	(±0.6)	(±13.4)	(±17.3)	(±3.5)	(±4.4)
*f*_3_	110.8	109.1	73.7	74.7	35.9	36.1	80.3	85.6	17.0	17.5
SD	(±11.6)	(±11.3)	(±11.5)	(±9.8)	(±0.6)	(±0.5)	(±14)	(±16.7)	(±5.8)	(±5.8)
*f*_4_	112.6	112.0	76.2	76.9	36.0	36.1	80.8	85.8	15.0	17.3
SD	(±13.0)	(±12.8)	(±8.5)	(±8.7)	(±0.4)	(±0.5)	(±10.6)	(±12.1)	(±3.1)	(±3.3)

**Table 2:** Mean and standard error (SE) for all 24 subjects in the normal condition. The values are the baseline measurements (“before WBV”) for each site on the tested foot (1: heel, 2: mid-foot, 3: 5th metatarsal head, 4: 1st metatarsal head, and 5: hallux).

		Touch [log⁡10 (1000F[N])] Right foot		Touch [log⁡10 (1000F[N])] Left foot	
		Mean	SE	Mean	SE
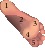	1	3.59	0.05	3.59	0.05
	2	3.08	0.07	3.05	0.07
	3	3.31	0.08	3.35	0.06
	4	3.33	0.08	3.27	0.09
	5	3.40	0.08	3.37	0.07
